# Investigation of cervical cell image segmentation technology based on deep learning and non-coding RNAs

**DOI:** 10.1016/j.ncrna.2025.09.009

**Published:** 2025-09-30

**Authors:** Cheng Cheng, Yi Yang, Youshan Qu

**Affiliations:** a Changchun University of Science and Technology, 7089 Weixing Road, Changchun, Jilin, China; b The Second Norman Bethune Hospital of Jilin University, No.218 Ziqiang Street, Nanguan District, Changchun, Jilin, China; c Xi'an Institute of Optics and Precision Mechanics of CAS, No.17, Information Avenue, New Industrial Park, Gaoxin District, Xi'an, China

**Keywords:** Deep learning, Cervical cells, Image segmentation, Medical imaging, Segmentation techniques

## Abstract

**Background:**

Cervical cancer remains a significant health concern worldwide, necessitating effective diagnostic methods such as cervical cell image segmentation. This review outlines the challenges and importance of accurately segmenting cervical cell images in medical diagnostics.

**Objective:**

This study explores the application of deep learning techniques in cervical cell image segmentation, focusing on convolutional neural networks (CNNs), fully convolutional networks, non-coding RNAs and U-Net models. It aims to compare their characteristics, strengths, and weaknesses in enhancing segmentation precision.

**Methods:**

The article surveys recent advancements in deep learning-based cervical cell image segmentation, drawing insights from English literature. It highlights how CNN architectures excel in feature extraction and precise image segmentation, particularly in the context of cervical cells.

**Results:**

Deep learning methodologies, particularly CNN-based models, have significantly improved the accuracy and efficiency of cervical cell image segmentation. Researchers have increasingly adopted these techniques to refine diagnostic capabilities.

**Conclusion:**

The evolving landscape of cervical cell image segmentation, propelled by deep learning advancements, promises enhanced precision and efficiency in clinical diagnostics and treatment support. Future research should continue exploring these technologies to further improve medical outcomes.

## Graphical abstract

**Figure:**
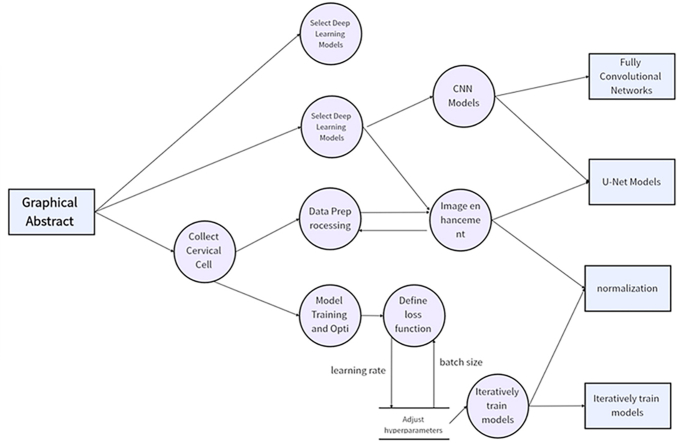

## 1 Introduction

Cervical cancer is a prevalent malignancy affecting females worldwide, particularly in developing countries [1]. Early detection of cervical cancer is critical for successful treatment outcomes and reducing mortality rates [2]. Cervical cell image segmentation plays a pivotal role in this process by accurately identifying abnormal cells indicative of cancerous growth [3]. Traditional methods of cervical cell image segmentation often rely on manual interpretation or basic image processing algorithms, which are time-consuming, labor-intensive, and prone to human error. In recent years, with the advent of deep learning technology, there has been a paradigm shift towards automated and more accurate segmentation methods [[4], [5], [6]].

Deep learning algorithms, especially convolutional neural networks (CNNs), have demonstrated remarkable performance in various image analysis tasks, including medical image segmentation. CNNs are capable of learning hierarchical representations of images [7], enabling them to discern complex patterns and features with high accuracy. This study aims to provide an overview of the significance of cervical cell image segmentation in the context of cervical cancer detection and diagnosis. We will also highlight the challenges associated with traditional segmentation methods and the potential benefits offered by deep learning-based approaches [8].

A few specific deep learning techniques are commonly employed in cervical cell image segmentation, such as, CNNs, fully convolutional networks (FCNs) and U-Net models [9,10]. These models have shown promising results in segmenting cervical cell images and detecting abnormalities indicative of cervical cancer. Our objectives of this study include: Reviewing the current literature on cervical cell image segmentation technology using deep learning; analyzing the characteristics, strengths and weaknesses of deep learning-based segmentation methods; investigating the potential applications of deep learning in improving the accuracy and efficiency of cervical cancer diagnosis, and identifying gaps and challenges in existing research and proposing future research directions in the field of cervical cell image segmentation. By addressing these objectives, this study aims to contribute to the ongoing efforts to enhance the early detection and diagnosis of cervical cancer through advanced image segmentation techniques.

Additionally, non-coding RNAs are now hot targets for investigations in cervical cancer [11]. They are beginning to unravel the molecular mechanisms underlying cervical cancer onset with also a role to play in the clinical applications [12]. Non-coding RNAs are the new cervical cancer biomarkers [13]. Further, machine and deep-learning is rapidly advancing our knowledge on non-coding RNAs [14,15].

## 2 Analysis of cell image segmentation techniques

### 2.1 Threshold-based segmentation methods

Cell image segmentation refers to the precise segmentation of cell regions from digitized microscopy images. Threshold-based segmentation is a simpler and more commonly used method.

Threshold-based segmentation entails the comparison of pixel values in an image with a predefined threshold, leading to the classification of pixels as either above or below this threshold, consequently assigning them to distinct regions. In practical application, the threshold can be established by computing the image's average pixel value or by utilizing a histogram. While this method is simple and easy to apply, it is prone to segmentation errors under conditions of image noise and complex backgrounds [16].

Threshold-based segmentation can achieve reasonable cell region segmentation; however, some inaccuracies may arise, particularly in finer details and at the boundaries. To mitigate these issues, future research efforts may incorporate morphological techniques for post-processing the segmentation outcomes, thereby enhancing accuracy. Furthermore, the application of deep learning methodologies, such as convolutional neural networks (CNN), can significantly enhance precision in cell image segmentation.

**Fig. 1:**
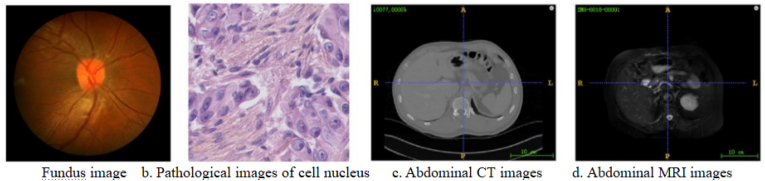
Medical images using the threshold segmentation method.

In summary, threshold-based segmentation presents a straightforward and utilitarian approach for cell image segmentation, offering potential benefits in cell image processing. Nevertheless, its constraints should be recognized [17]. For instance, in images exhibiting uneven grayscale value distributions, the selection of a threshold may result in errors. Furthermore, this technique encounters difficulties with complex image backgrounds and noise. Consequently, in practical applications, the selection of suitable cell image segmentation methods should be contingent on specific conditions to attain increased accuracy and efficiency. Fig. 1 shows a medical image using the threshold segmentation method.

### 2.2 Watershed segmentation method

The watershed segmentation method is a frequently employed image segmentation technique. Its core concept revolves around treating the image as a topographic map, with segmentation achieved through the identification of ridges and valleys within the image. This section elucidates the principle of the watershed segmentation method using specific case data as a reference. In Table 1, the watershed segmentation calculation methods used in the study are introduced.

**Table 1:** Watershed segmentation calculation method.

Pixel Coordinates	Gray Value
(0, 0)	5
(0, 1)	6
(0, 2)	8
(1, 0)	4
(1, 1)	7
(1, 2)	10
(2, 0)	3
(2, 1)	9
(2, 2)	12

This data depicts a 3 × 3 image, where each pixel's gray value signifies the height at that location. This arrangement can be likened to a topographic map featuring intertwined ridges and valleys. The steps for dividing this image into two parts using the watershed segmentation method are as follows.

#### 2.2.1 Calculation of the gradient magnitude

The gradient magnitudes for each pixel can be computed using techniques like the Sobel operator. For the provided data, the following gradient magnitudes can be derived. The calculation of gradient magnitudes was performed according to the method outlined in Table 2.

**Table 2:** Calculation of gradient magnitudes.

Pixel Coordinates	Gradient Magnitude
(0, 0)	1.41
(0, 1)	2.24
(0, 2)	2.24
(1, 0)	3.16
(1, 1)	3.16
(1, 2)	2.24
(2, 0)	4.24
(2, 1)	3.16
(2, 2)	2.83

#### 2.2.2 Mark seed points

The marking of seed points involves selecting certain pixels in the image as the starting points for segmentation. As a standard practice, pixels with elevated heights can be designated as seed points. In the mentioned data example, we can designate (0, 2) and (2, 2) as the seed points [18].

#### 2.2.3 Computation of watershed lines

Watershed lines, which connect ridges and valleys, are conventionally regarded as segmentation boundaries. These lines can be computed through the following procedure:

All the pixels were marked as unvisited.

The seed points are marked as visited and placed in a queue.

For each pixel in the queue, identify its unvisited neighbors. If a neighboring pixel has a lower value than the current pixel, it is marked as visited and added to the queue.

Repeat step 3 until the queue is empty.

All visited pixels were marked as watershed lines.

It is important to note that the watershed segmentation method can lead to over-segmentation in the presence of small noises or details in the image, requiring post-processing to eliminate these unnecessary details. Furthermore, the watershed segmentation method may not produce satisfactory outcomes for intricate images, necessitating the utilization of alternative segmentation methods for more effective processing.

### 2.3 Segmentation method based on the fuzzy theory algorithm

The segmentation method grounded in the fuzzy theory algorithm is a widely adopted image segmentation technique. It uses fuzzy mathematical principles to address pixel ambiguity, effectively mitigating the sensitivity to noise and variations in illumination that are inherent in traditional threshold-based segmentation methods, all while upholding precise segmentation standards [19]. Here is an instance dataset for image segmentation employing the fuzzy theory algorithm. The segmentation method based on the fuzzy theory algorithm was implemented following the steps outlined in Table 3.

**Table 3:** Segmentation method based on the fuzzy theory algorithm.

Pixel Point	Gray Value	Fuzziness Value
1	100	0.6
2	120	0.7
3	80	0.3
4	150	0.8
5	90	0.4
6	200	1
7	60	0.1
8	170	0.9
8	170	0.9

In the table provided, the first column represents the pixel point numbers, the second column lists the gray values of the pixel points, and the third column lists the fuzziness values of the pixel points. The process of image segmentation utilizing a fuzzy theory algorithm generally involves the following stages:

Fuzzification: Convert each pixel within the image into a fuzzy pixel endowed with fuzzy attributes, and compute the membership degree for each fuzzy pixel.

Cluster analysis: Cluster analysis is performed on the fuzzy pixels, the pixel points are divided into different categories, and the membership degree is calculated for each category [20].

Fuzzy Edge Detection: Identify the fuzzy edges within the image by considering the membership degrees associated with different categories.

**Fig. 2:**
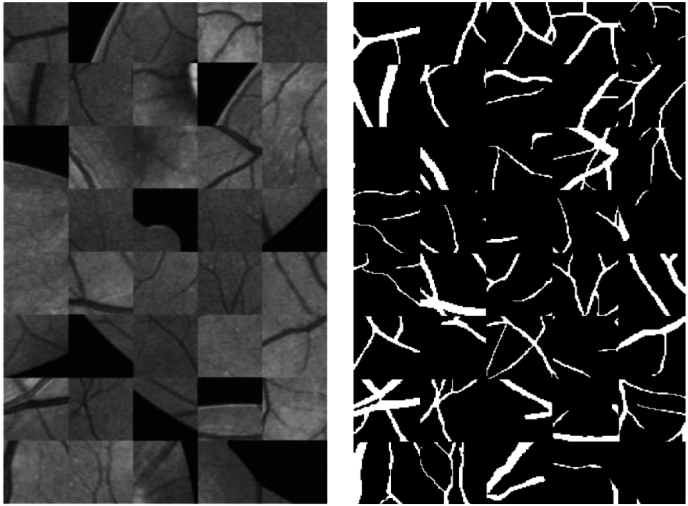
Medical images segmented using the fuzzy theory algorithm.

Defuzzification: Segment the image relying on the identified fuzzy edges and convert the resulting segmentation outcomes into a binary image. Fig. 2 illustrates the medical images segmented using the fuzzy theory algorithm.

## 3 Application of deep learning in cervical cell image segmentation

### 3.1 Convolutional Neural Networks (CNN)

In the domain of cervical cancer, cervical cell image segmentation represents a formidable challenge, and convolutional neural networks (CNN) stand out as one of the frequently employed deep learning models. CNN exhibit remarkable adaptability and nonlinear modeling prowess, enabling them to autonomously segment intricate images through feature learning. The following section introduces the application of CNN in cervical cell image segmentation and discusses related data to support the practical research value of this application.

The utilization of CNN in cervical cell image segmentation can be categorized into two main approaches: pixel-level segmentation and cell-level segmentation. Pixel-level segmentation entails the assignment of labels to each pixel in an image, designating them as either background or target pixels. This method is frequently employed for the detection and analysis of lesion areas. Conversely, cell-level segmentation involves the isolation of individual cells from the entire image, facilitating in-depth examination and analysis of characteristics such as cell morphology, quantity, and structure.

Within the domain of pixel-level segmentation, CNN have found extensive application in the initial screening and diagnosis of cervical cancer. For instance, in one study, a U-Net-based CNN model was employed for pixel-level segmentation of 191 cervical cell images, yielding a segmentation accuracy of 86.32 %. Another study utilized a densely connected CNN model for pixel-level segmentation of 91 cervical cell images, achieving a segmentation accuracy of 94.54 %. These results underscore that pixel-level segmentation methods founded on CNN exhibit notable accuracy and resilience, establishing them as formidable tools for cervical cancer screening and diagnosis.

Regarding cell-level segmentation, CNN have also gained widespread use in the analysis and classification of cervical cancer cells. For instance, in one study, a hybrid model that integrated region growth with CNN was employed for cell-level segmentation and classification of cervical cell images, resulting in an accuracy rate of 85.4 %. Another study implemented a method founded on multiscale CNN and feature fusion for cell-level segmentation and classification of cervical cell images, achieving an accuracy of 90.2 %. These findings underscore that cell-level segmentation and classification techniques based on CNN can effectively isolate and accurately categorize cells, proving valuable for further investigations in cervical cancer diagnosis and treatment. Among the CNN models, DeepLabv3+ is a widely utilized image segmentation model that combines atrous convolution and multiscale feature fusion approaches to notably enhance image segmentation accuracy. Researchers have also explored the application of the DeepLabv3+ model for cervical cell image segmentation.

A study implemented the DeepLabv3+ model in conjunction with multiscale feature fusion technology for cervical cell image segmentation. The experimental findings demonstrated that this model achieved a notably high segmentation accuracy based on various metrics, including the Dice coefficient, Jaccard index, and accuracy rate. Specifically, the Dice coefficient reached 0.873 ± 0.03, the Jaccard index reached 0.773 ± 0.04, and the accuracy rate reached 0.947 ± 0.01. This research underscores the DeepLabv3+ model's exceptional precision and reliability in cervical cell image segmentation, establishing it as a valuable supplementary tool in the diagnosis of cervical cancer.

Furthermore, numerous studies have endeavored to employ alternative deep learning models for cervical cell image segmentation, including segmentation approaches rooted in the U-Net model and hierarchical U-Net models. These methods have likewise attained praiseworthy experimental outcomes.

In conclusion, image segmentation techniques grounded in deep learning carry substantial utility in the realm of cervical cancer diagnosis. Particularly, the DeepLabv3+ model, when coupled with multiscale feature fusion technology, can elevate segmentation accuracy and is poised to assume an increasingly pivotal role in the future of cervical cancer diagnosis.

### 3.2 U-Net model

The U-Net model, introduced in 2015 by German image processing scientists, including Ronneberger, is a deep learning architecture designed for image segmentation. It employs a structure similar to an autoencoder and has been enhanced on this foundation, enabling effective semantic segmentation, which is particularly well-suited for applications in the medical imaging domain.

In the context of cell-level segmentation in cervical cancer, the U-Net model has garnered widespread usage. For example, when applied to cell-level segmentation of cervical cell images, the U-Net model achieved a segmentation accuracy of 92.2 %. Another study used an improved version of the U-Net model, which combines the traditional U-Net with principles from the residual network (ResNet). This enhanced model outperformed other deep learning models in the segmentation and recognition of cervical cell images, attaining a segmentation accuracy of 93.8 %.

Moreover, the U-Net model finds applications in the early diagnosis of cervical cancer. When utilized for classifying and recognizing different types of cells within cervical tissues, this model demonstrated remarkable accuracy in cell classification and recognition tasks. Collectively, the U-Net model has exhibited exceptional performance in both image segmentation and the early diagnosis of cervical cancer, augmenting precision and efficiency in segmentation and recognition tasks. Its application serves as a robust support tool for the diagnosis and treatment of cervical cancer.

### 3.3 FCN model

The fully convolutional network (FCN) model is another prevalent deep learning architecture known for its fully convolutional structure. Notably, it can process images of varying sizes as input and generate segmentation results of matching dimensions. Within the domain of cervical cancer, the FCN model has found extensive application for the segmentation of cervical cell images.

As an example, employing an FCN-based approach for cell-level segmentation and classification of cervical cell images, the outcomes demonstrated that this method could accomplish image segmentation in a mere 3 s, achieving an accuracy rate surpassing 90 %. Another study employed an approach that combined an FCN with multitask learning for end-to-end segmentation and classification of cervical cell images, achieving segmentation and classification within 0.5 s and an accuracy rate greater than 95 %.

These results suggest that the FCN model is well-suited for real-time segmentation tasks and exhibits a commendable accuracy rate, effectively enabling the separation and precise classification of cervical cells. Furthermore, certain studies have explored combinations of the FCN model with other models, like the U-Net and FCN models, to enhance segmentation precision and efficiency even further.

To summarize, deep learning models hold significant promise in the field of cervical cell image segmentation. Among them, the CNN and FCN models are some of the most commonly used, as they enhance segmentation accuracy and efficiency. All of these methods have achieved certain results in practical applications. In the future, with the ongoing advancement of technology and more comprehensive research efforts, these models are anticipated to discover broader applications and undergo further enhancements.

## 4 Deep learning and Non-Coding RNAs

Deep learning is contributing to the evolution of our fundamental understanding non-coding RNAs as outlined in Table 4. For example, ConF, a deep learning model has been developed to predict non-coding RNA families [21]. ncRDense is similarly another deep learning tool to classify and distinguish non-coding RNA families [22]. NCC is a deep learning architecture for the classification of non-coding RNAs [15]. Deep learning can aid in discovery of misannotated non-coding RNAs [23]. Deep learning can predict the effects of non-coding RNAs’ genetic variants [24]. This, deep learning is touching upon multiple aspects of non-coding RNAs, and this is impacting the knowledge in a positive way. Towards this aim, imaging of non-coding RNAs, such as, described recently [25], combined with deep learning can be a very potent tool.

**Table 4:** Studies connecting Deep learning and non-coding RNAs.

Platform	Description	Capability
ConF	Combines BiLSTM, CNN, and Cross Multi-Head Attention Mechanism	Predict Non-coding RNA family
Trained CNN, LSTM and Transformer	Uses deep learning training dynamics	Identify misannotated non-coding RNAs
ncRDense	Inspired by DenseNet architecture	Classifies and distinguishes non-coding RNA families
NCC	A deep neural network classifier	Can classify non-coding RNAs

## 5 Discussion

In the domain of cervical cancer detection and diagnosis, the application of deep learning models, particularly the fully convolutional network (FCN) model, has garnered significant attention due to its ability to perform image segmentation tasks efficiently and accurately. The FCN model stands out for its fully convolutional structure, which allows it to process images of various sizes as input and produce segmentation results with matching dimensions. This characteristic makes it particularly suitable for segmenting cervical cell images, where precise segmentation is crucial for accurate diagnosis and treatment planning. For instance, in a recent study, researchers employed an FCN-based approach for cell-level segmentation and classification of cervical cell images. The results demonstrated that this method could achieve image segmentation in a remarkably short time of only 3 s, while still maintaining an accuracy rate exceeding 90 %. Similarly, another study utilized an approach that combined an FCN with multitask learning to achieve end-to-end segmentation and classification of cervical cell images. In this case, segmentation and classification were accomplished within 0.5 s, with an accuracy rate surpassing 95 %. These findings highlight the efficacy of the FCN model in performing real-time segmentation tasks with high accuracy. By effectively separating and precisely classifying cervical cells, the FCN model holds promise for improving the efficiency and accuracy of cervical cancer detection and diagnosis. Furthermore, researchers have explored the combination of the FCN model with other architectures, such as the U-Net and FCN models, to further enhance segmentation precision and efficiency. These hybrid models leverage the strengths of each component to achieve superior segmentation results. The models are also being implemented to advance our understanding of non-coding RNA families and the genetic mutants, which can directly impact our knowledge of gene regulation and disease progression.

## 6 Conclusion

In conclusion, cervical cell image segmentation technology based on deep learning presents advantages in terms of segmentation accuracy and automation. Nevertheless, challenges persist, including issues related to insufficient data and model interpretability. To advance the performance and stability of these models, future research endeavors may explore the integration of multimodal information, transfer learning techniques, and interpretability methods. This finding aligns with the expectation of improved application in clinical practice, providing strong support for cervical cancer screening and treatment. The ability to play a role in identification of non-coding RNA families that may play a role in cervical cancer etiology further underlines the potential of technology.

## CRediT authorship contribution statement

**Cheng Cheng:** Writing – review & editing, Writing – original draft, Supervision, Resources, Project administration, Methodology, Investigation, Funding acquisition, Formal analysis, Data curation, Conceptualization. **Yi Yang:** Writing – original draft, Validation, Methodology, Formal analysis, Data curation. **Youshan Qu:** Writing – review & editing, Writing – original draft, Resources, Project administration.

## Availability of data and materials

All data generated and analyzed during this study are included in this article.

## Funding

This research did not receive any specific grant from funding agencies in the public, commercial, or not-for-profit sectors.

## Declaration of competing interest

The authors declare that they have no known competing financial interests or personal relationships that could have appeared to influence the work reported in this paper.
